# Manifestations and Treatment of Hypovitaminosis in Oral Diseases: A Systematic Review

**DOI:** 10.3390/dj12060152

**Published:** 2024-05-21

**Authors:** Barbara Bačun, Dora Galić, Luka Pul, Matej Tomas, Davor Kuiš

**Affiliations:** 1 Faculty of Dental Medicine and Health of Osijek, Josip Juraj Strossmayer University of Osijek, Crkvena 21, 31 000 Osijek, Croatia; bbacun@fdmz.hr (B.B.); dgalic1@fdmz.hr (D.G.); 2 Community Healthcare Center of Osijek-Baranja County, 31 000 Osijek, Croatia; lukapul11@gmail.com; 3 Department of Dental Medicine, Faculty of Dental Medicine and Health of Osijek, Josip Juraj Strossmayer University of Osijek, Crkvena 21, 31 000 Osijek, Croatia; 4 Department of Periodontology, Faculty of Dental Medicine Rijeka, University of Rijeka, 51 000 Rijeka, Croatia; 5 Clinical Hospital Center Rijeka, 51 000 Rijeka, Croatia

**Keywords:** oral cavity, oral manifestations, vitamin deficiency

## Abstract

This review’s objective is to examine the findings from various studies on oral signs and symptoms related to vitamin deficiency. In October 2023, two electronic databases (Scopus and PubMed) were searched for published scientific articles following PRISMA principles. Articles eligible for inclusion in this review had to be published in English between 2017 and 2023, be original studies, and involve human subjects. Fifteen studies were included in this review: three examining oral symptoms of vitamin B12 deficiency; one assessing vitamin B complex and vitamin E for recurrent oral ulcers; one investigating serum vitamin D levels in recurrent aphthous stomatitis patients; three exploring hypovitaminosis effects on dental caries; two measuring blood serum vitamin D levels; one evaluating vitamin B12 hypovitaminosis; three investigating hypovitaminosis as indicative of gingival disease; one focusing on vitamin deficiencies and enamel developmental abnormalities; one assessing vitamin deficiencies in oral cancer patients; one examining vitamin K as an oral anticoagulant and its role in perioperative hemorrhage; and one evaluating vitamin effects on burning mouth syndrome. Despite some limitations, evidence suggests a correlation between vitamin deficiencies and oral symptoms. This systematic review was registered in the International Platform of Registered Systematic Review and Meta-analysis Protocols (INPLASY) database (202430039).

## 1. Introduction

Vitamins are a group of organic compounds essential for normal metabolic reactions. Most vitamins cannot be synthesized by humans and must be obtained from food, while others are produced by the gut microbiome. Vitamins can be divided into two groups: water-soluble and fat-soluble. The water-soluble vitamins that are most important for oral health are vitamin C (ascorbic acid) and B complex vitamins: B2 (riboflavin), B3 (niacin), B6 (pyridoxine), B9 (folic acid), and B12 (cobalamin). Fat-soluble vitamins that have a significant effect on the oral mucosa are vitamin A (retinol), vitamin D (calciferol), vitamin K (phylloquinone), and vitamin E (tocopherol) [1]. For optimal functioning of the human body, a balance between supplied vitamins is required; if homeostasis is disturbed, vitamin deficiency occurs.

Hypovitaminosis or vitamin deficiency may result from decreased vitamin intake or impaired absorption [2]. Most vitamin deficiencies can be recognized by obvious clinical syndromes that occur in areas of the world with poor nutrition; in resource-rich societies, they occur in various populations, including older adults, vegans, people living in poverty, patients with alcohol consumption, malabsorption disorders, limited sun exposure, or inborn abnormalities, and people undergoing hemodialysis [3].

We have included in this review all significant material released in the last six years to summarize recently acquired data in this field of study. Given the insufficiency of systematic reviews addressing this specific topic, our decision to undertake this review was motivated by its perceived significance for informing future research and clinical application. Through this systematic review, we aim to provide valuable insights for clinicians and researchers to enhance oral health promotion and disease prevention efforts, particularly in populations vulnerable to nutritional deficiencies. Ultimately, this review seeks to underscore the importance of addressing hypovitaminosis as a modifiable risk factor in oral disease management and improving overall oral health outcomes. The objective of this review is to assess recent findings regarding hypovitaminosis concerning oral health and the use of vitamins in the treatment of oral disease.

### 1.1. Vitamin A

Vitamin A alters the activity of multiple cell lines, can stimulate epithelial healing, and plays an important role in all stages of wound healing. A lower intake of vitamin A can lead to a variety of cutaneous manifestations and has been associated with decreased development of oral epithelium, impaired tooth formation, enamel hypoplasia, and periodontitis. Dietary sources of vitamin A include eggs, cod liver oil, carrots, capsicum, liver, sweet potatoes, broccoli, and leafy vegetables. A healthy individual needs approximately 900 µg/day [2,4].

Due to rapid cell growth and epithelial cell turnover in the mucous membranes, which may affect some or all the tissues of the oral cavity (including the teeth, periodontal tissues, salivary glands, mucous membranes, and perioral skin), the oral cavity is a specific anatomic area that may exhibit early signs of nutritional disorders [3].

### 1.2. Vitamin B

Vitamin B complex is a group of eight compounds (B1, B2, B3, B5, B6, B7, B9, and B12) that are necessary for cell metabolic activities and normal functions [1].

The proximal small intestine is the primary site of absorption of vitamin B2 (riboflavin). In recent years, its deficiency has been relatively uncommon and is restricted to elderly patients exhibiting dermatitis of the anogenital area, severe photophobia correlated with ocular vascularization, and seborrheic follicular keratosis of the forehead and nose. [1,5]. Riboflavin deficiency can cause a variety of oral symptoms, such as angular cheilitis, recurring aphthous stomatitis, and edema of the pharynx and oral mucous membrane. Atrophic, riboflavin-deficient glossitis is frequently described as having a magenta tint. The atrophied filiform papillae may be covered by fungiform papillae that have grown larger [6].

Vitamin B3 (niacin) is a crucial component of the oxidation–reduction reaction. Niacin deficiency leads to pellagra, a condition characterized by dementia, dermatitis, and inflammation of the mucus membranes of the intestines. Patients may experience a prodromal burning in the mouth before the oral symptoms of pellagra. Oral symptoms include tongue swelling and redness, buccal mucosa ulcers across from the molars, and a thick, gray discharge of degenerating cells that may create a pseudomembrane on the dorsal tongue. In addition to burning mouth syndrome, vitamin B3 hypovitaminosis is also associated with dental caries, angular cheilitis, severe pain, and redness of the gingiva [3,6].

Heme biosynthesis, fatty acid metabolism, gluconeogenesis, and the production of neurotransmitters are all regulated by vitamin B6 (pyridoxine). Scaling of the skin, digestive issues, and other health issues can all result from a vitamin deficiency, specifically in areas around the nose and lips, and oral epithelial changes. Examples of oral manifestations include angular stomatitis, cheilitis, atrophic glossitis, and gingival erythema [7].

Oral epithelial cell turnover is dependent on folic acid because vitamin B9 (folate) is a coenzyme cofactor in DNA synthesis [3]. The most common symptom of folate deficiency is megaloblastic anemia, which is defined by a defect in the development of erythropoietic cell precursors and the formation of large, irregular nucleated erythrocytes in the bone marrow. High cellular turnover, which causes glossitis, is the cause of oral symptoms linked to megaloblastic anemia. Due to the significance of folate in preserving DNA integrity, diets low in folate may also be linked to an increased risk of orofacial clefts, oral cavity cancer, and pharyngeal cancer. There is a correlation between oral lichen planus occurrence and iron, folic acid, and vitamin B12 deficiency [8].

Vitamin B12 (cobalamin) is also a crucial cofactor in the synthesis of DNA [3]. Pernicious anemia, the most common form of vitamin B12 deficiency, is caused by autoantibodies against intrinsic factor (IF) and/or stomach parietal cells and may be hereditarily predisposed [9]. Megaloblastic anemia, which is comparable to folate deficiency, is the outcome of vitamin B12 deficiency. Generalized stomatitis, taste difficulties, and a red, atrophic, meaty, burning tongue with a bald appearance because of the absence of filiform papillae are all symptoms of cobalamin insufficiency [4,10]. One of the oral symptoms is a prodrome of burning and discomfort of the tongue. Reduced taste is caused by fissuring and probable circumvallate papillae loss. Primary burning mouth syndrome is occasionally mistaken for Hunter’s glossitis, a painful, atrophic, and red tongue brought on by a vitamin B12 deficiency [11].

### 1.3. Vitamin C

Vitamin C (ascorbic acid) is essential for the synthesis of collagen, immunological processes involved in wound healing, and possibly the repair of periodontal tissues [12,13]. Studies have shown its potential protective effects in oral cancers, with decreased saliva levels observed in patients compared to controls. Dietary intake of vitamin C has been associated with a reduced risk of oral premalignant lesions, while a high intake from natural sources correlates with a lower risk of head and neck cancer. Furthermore, oral cancer patients exhibit marked decreases in vitamin C levels, suggesting its deficiency as a risk factor for carcinogenesis. Consequently, vitamin C is recommended as a therapeutic measure to mitigate oral cancer initiation and progression [14]. Beyond its role in cancer prevention, vitamin C is indispensable for collagen synthesis, crucial for wound healing, and potentially important for periodontal tissue repair. Deficiency in vitamin C can lead to gingival changes, gingivitis, and periodontitis. Moreover, severe deficiency can result in scurvy, a life-threatening condition requiring vitamin C administration. Patients with scurvy may experience delayed healing, xerostomia, and reduced neutrophil chemotaxis, highlighting the essentiality of adequate vitamin C intake for oral health and overall well-being [15].

### 1.4. Vitamin D

Vitamin D has an antibacterial effect by reducing cell proliferation and promoting differentiation. It also regulates immunological functions, calcium phosphate metabolism, and calcium metabolism [16]. It has been linked to a wide variety of oral health disorders. Severe vitamin D hypovitaminosis in children can lead to abnormal mineralization of the teeth, resulting in dentin and enamel defects. A lack of vitamin D can result in enamel and dentin hypoplasia, dysplasia, and non-syndromic amelogenesis and dentinogenesis imperfecta during tooth development. As a result of these defects, a more rapid progression of dental caries can occur. Additionally, gingival inflammation and a higher prevalence of periodontitis are linked to vitamin D deficiency. Low vitamin D levels have also been connected to bone remodeling, tissue regeneration, squamous oral cancer therapy, and recurrent aphthous stomatitis [16,17].

### 1.5. Vitamin E

An umbrella term, vitamin E is used to describe a family of eight plant-derived, fat-soluble compounds (α-, β-, γ-, δ-Ts, and α-, β-, γ-, and δ-tocotrienols). Vitamin E prevents lipid peroxidation in biological membranes by operating as a free radical neutralizer. In addition to its typical function as an antioxidant, vitamin E also influences gene and protein expression, signaling cascades, and the function of enzymes [18]. Vitamin E can be found primarily in food sources such as soybeans, rapeseed, wheat germ, sunflower, and corn germ oils [19]. Patients suffering from conditions that limit the absorption of fat-soluble nutrients and dietary fats frequently develop vitamin E deficiency. Studies revealed no significant differences in plasma vitamin E levels between healthy individuals and those with periodontitis. Conversely, some investigations reported beneficial effects of vitamin E in preserving periodontal health and managing inflammation. Moreover, reduced vitamin E levels were noted in periodontal disease patients compared to healthy counterparts. Smokers exhibited decreased levels of vitamin E in alveolar fluid, likely attributable to heightened oxidant production during smoking. Despite these findings, the precise mechanism underlying vitamin E’s role in periodontal health remains incompletely elucidated and warrants further investigation [20].

### 1.6. Vitamin K

Vitamin K is a group of vitamins required for the synthesis of proteins that are precursors or prerequisites of the formation of blood coagulation factors such as prothrombin and factors VII, IX, and X [15]. Patients undergoing oral surgery or experiencing an oral injury may be affected by a vitamin K deficiency [1,21].

## 2. Materials and Methods

### 2.1. Protocol and Registration

This review uses preferred reporting items for systematic reviews, meta-analysis (PRISMA) guidelines, and PICO criteria (Table 1 and Table 2).

This systematic review was registered in the International Platform of Registered Systematic Review and Meta-analysis Protocols (INPLASY) database (202430039). The DOI number is 10.37766/inplasy2024.3.0039.

### 2.2. Literature Search Strategy

A comprehensive search strategy was devised by two authors (B.B. and D.G.) to identify the relevant literature pertaining to the association between vitamin deficiencies and oral diseases. The search was conducted at the National Medical Library from the 8th to the 15th of October 2023, utilizing the electronic databases PubMed and Scopus, renowned repositories for biomedical research articles. Both Medical Subject Headings (MeSH) and specific keywords were employed to maximize the comprehensiveness of the search.

The search strategy comprised the following keywords and MeSH terms, each paired with variations indicating both the deficiency of the specific vitamin and its association with oral diseases:

- Vitamin A deficiency and oral disease;
- Vitamin B deficiency and oral disease;
- Vitamin C deficiency and oral disease;
- Vitamin D deficiency and oral disease;
- Vitamin E deficiency and oral disease;
- Vitamin K deficiency and oral disease;
- Vitamin deficiency and oral disease.

### 2.3. Eligibility Criteria

The evaluation covered cohort studies, case–control studies, longitudinal studies, retrospective studies, controlled clinical trials, randomized controlled trials, and cross-section studies.

The following criteria had to be met for an article to be eligible for this review:

- Articles published in English;
- Research published between 2017 and 2023;
- Original studies;
- Studies conducted on human subjects.

Criteria for rejection of articles were as follows:

- Articles not written in English;
- Papers published before 2017;
- Case reports;
- Studies conducted on animals.

### 2.4. Selection Process

Three reviewers (B.B., D.G., and M.T.) first examined the titles and abstracts of the gathered publications. The texts of the studies that appeared to fit the inclusion criteria were gathered and examined separately. Eventually, the eligibility of a study was determined by using the inclusion and exclusion criteria. Disagreements among the reviewers were discussed and decided throughout the process of choosing the studies.

### 2.5. Data Collection Process

All articles that were retrieved had their titles and abstracts independently checked by the researchers. From the chosen, qualified articles, full-text data were extracted. After double-checking for accuracy, the extracted data were compared.

The following data were extracted from the eligible studies: number of participants, age range of participants, intervention/exposure, comparator/control, outcomes of interest, and main results.

### 2.6. Data Items

The insufficient amount of data and a high degree of heterogeneity among the qualifying studies precluded the performance of a statistical synthesis in the form of a meta-analysis.

Meta-analysis was not performed due to several reasons. Firstly, the included studies exhibited significant heterogeneity in methodology, population, and interventions, rendering meta-analysis inappropriate. Secondly, data insufficiency, such as missing statistical parameters required for quantitative analysis, precluded meta-analysis feasibility. Lastly, the research objectives did not necessitate meta-analysis, as the focus was on describing the diversity of results among studies and addressing qualitative aspects of the research questions.

We did not conduct a meta-analysis; therefore, we did not perform sensitivity analyses for combined results. Instead, we conducted individual analyses for each study to assess their reliability and impact on overall conclusions.

### 2.7. Risk of Bias Assessment

Risk of bias (RoB) was conducted according to the current versions of Cochrane RoB 2, Risk of Bias in Non-randomized Studies-of Interventions (ROBINS-I), and the Newcastle–Ottawa Scale (NOS) [22,23,24]. For each study, the most appropriate of the three tools was chosen based on the specific study design.

The following criteria were used to evaluate the study quality following the revised Cochrane risk-of-bias tool for randomized trials (RoB 2): (D1) randomization process bias; (D2) deviation bias from planned interventions; (D3) missing outcome data bias; (D4) outcome measurement bias; and (D5) reported outcome selection bias. For each of the included studies, the authors independently applied the tool and documented supporting data to evaluate the overall risk of bias in each category (low risk; some concerns; high risk).

Using the ROBINS-I tool, the quality of the studies was assessed based on the following criteria: (D1) bias due to confounding; (D2) bias in selection of participants for the study; (D3) bias in classification of interventions; (D4) bias due to deviations from intended interventions; (D5) bias due to missing data; (D6) bias in measurement of outcomes; (D7) bias in selection of the reported result. Each particular result was given an overall risk-of-bias rating (low risk of bias; moderate risk of bias; serious risk of bias; critical risk of bias; no information) based on the ROBINS-I guidance.

The Newcastle–Ottawa Scale (NOS) consists of eight items with three subscales; the combined maximum score of these three subsets is nine. We categorized any study with a score of at least seven as high-quality because there is not yet agreement on what constitutes a high-quality study.

### 2.8. Effect Measures

Each study utilized different measures based on the specific outcomes they were investigating. These include mean differences, risk ratios, odd ratios, correlation coefficients, proportions, and reductions, depending on the nature of the data and the hypothesis being tested.

Studies on diseases of the oral mucosa focused on the effects of vitamin deficiencies, specifically vitamin B12, on conditions including geographic tongue, lingual linear lesions (LLL), atrophic glossitis, and recurrent aphthous stomatitis (RAS). The serum levels of vitamins and their correlations with oral symptoms were evaluated using effect measures such as mean differences and risk ratios.

The association between vitamin deficiencies, especially those of vitamin B12 and vitamin D, and the occurrence of dental caries was evaluated in dental caries research using mean differences and indexes such as decay-missing-filled teeth.

Proportion measurements and reductions have been used in studies on gingival and periodontal disease to evaluate the impact of vitamin deficiencies—especially those of vitamin C and vitamin D—on gingivitis and periodontitis.

Odds ratios have been used in malformation studies to evaluate the connection between children’s enamel abnormalities and their mothers’ vitamin D levels.

Mean differences, risk ratios, and visual analog scales were used in additional research on oral cancer, perioperative bleeding, and burning mouth syndrome to evaluate the impact of vitamin deficiencies on disease incidence and symptom severity.

### 2.9. Compliance with Ethical Guidelines

This article is based on studies that have already been published. Subsequently, there are no ethical issues with the study.

## 3. Results

### 3.1. Study Selection

**Figure 1:**
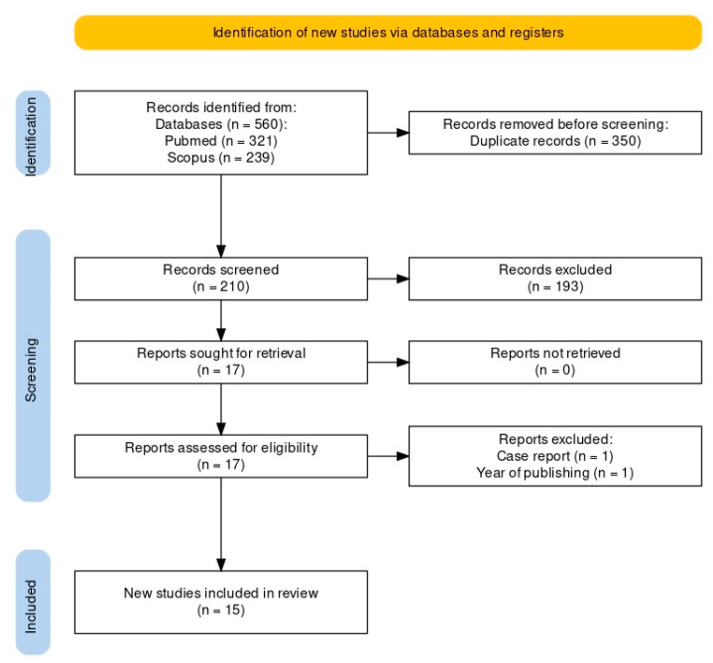
Prisma flowchart of search results.

Before screening, duplicated articles (350 entries) were removed. Titles and abstracts were used to filter a total of 210 articles. Articles that were not relevant to this systematic review were excluded after titles and abstracts were evaluated (193 entries). Seventeen articles were selected as a result of full-text analysis. Fifteen studies were included in this study after two articles (one due to the year of publishing and the other being a case report) were eliminated. A flowchart of the study selection process is shown in Figure 1.

### 3.2. Risk-of-Bias Assessment

Table 3, Table 4 and Table 5 present the results of the Risk-of-Bias evaluation. Using the RoB 2 tool (version 2), four randomized trials were assessed. One study was found to raise some issues in domains D1, D2, D4, and D5. These issues included missing explicit information about the study’s pre-specified analysis plan, failing to provide clear explanations for any missing outcome data, and lacking specific information about the study’s allocation process. The Newcastle–Ottawa Scale (NOS) was used to evaluate cross-sectional studies, whereas ROBINS-I was used to evaluate case–control, longitudinal, retrospective, and cohort studies.

### 3.3. Nature of Included Studies

Data collected from each study included the following: four randomized controlled studies, seven cross-section studies, one case–control study, one retrospective study, one longitudinal study, and one cohort study. Table 6 provides a summary of the included studies, describing each hypovitaminosis’s oral manifestation. The table is divided into the following categories: author, title, year of publication, type of study, oral manifestation, number of participants, age range of participants, intervention/exposure, comparator/control, vitamin, outcomes of interest, and main results.

### 3.4. Diseases of the Oral Mucosa

Five studies included in this review evaluated the effect of vitamins on soft oral tissues. Three studies examined oral manifestations of vitamin B12 hypovitaminosis (recurrent aphthous stomatitis, atrophic glossitis, lingual linear lesions, and geographic tongue), one study included vitamin B complex and vitamin E in the treatment of recurrent oral ulcer, and one study measured serum vitamin D in patients with recurrent aphthous stomatitis.

Wu, Y.-H. et al. [33] compared collected blood count and serum levels of vitamin B12, folate, and ferritin of 63 patients with recurrent aphthous stomatitis (RAS) with 126 healthy subjects, and they exhibited considerably reduced mean serum levels of folic acid and vitamin B12 compared to healthy subjects. A total of 30 out of 63 patients with RAS had atrophic glossitis (AG+) and had significantly greater rates of folic acid and vitamin B12 deficiencies compared to healthy subjects, while 3 out of 63 patients with RAS had no atrophic glossitis (AG-) and had significantly higher rates of vitamin B12 deficiencies compared to healthy subjects. Anemia was present in 19 out of 63 RAS patients (13 AG+/RAS and 6 AG-/RAS). The most prevalent kind of anemia was normocytic anemia.

A retrospective study conducted by Bao, Z. et al. [29] investigated the incidence of lingual linear lesions (LLLs) with vitamin B12, folate, ferritin, and zinc levels. A total of 35 out of 57 patients presented with LLLs were in the AG-positive, and 22 were in the AG-negative group. The mean level of vitamin B12 in the AG+/LLL group was considerably lower than in the AG-/LLL group. Furthermore, 56 out of 57 patients had severe serum vitamin B12 insufficiency (no significant differences between groups), with 1 patient having a borderline low level of the vitamin. Additionally, anemia was identified in 13 (22.81%) patients. After the therapy, 30 out of 57 patients (52.63%) reported improvement in oral symptoms and general condition within the first three days, and after one month, all returned to the reference range.

Khayamzadeh, M. et al. [30] conducted a case–control study comparing vitamin B12 levels in blood serum and saliva of 20 patients with geographic tongue and 20 healthy individuals. No statistically significant difference in the serum levels of vitamin B12 was found.

The benefits of vitamin B12 and vitamin E combined in the treatment of recurrent oral ulcers were examined in the study by Cui, J. et al. [25] on 58 patients; 29 received mecobalamin 0.5 mg and vitamin E 200 mg soft capsules (test group), while 29 received Fe complex enzyme gargle (control group). The following parameters were measured: pain levels using a 10-point visual analog scale (VAS), ulcer status and number of ulcers, proinflammatory cytokine levels, and quality of life using a 36-item Short Form Health Survey (SF-36) from before and after the treatment. Total intermission time was significantly longer, mean ulcer healing time was considerably shorter, and the total number of ulcers was lower in the test group. VAS scores, interleukin 8 (IL-8) tumor necrosis factor alpha, and C-reactive protein were appreciably down-regulated in both groups when compared before and after treatment. The overall efficacy rate after one month of therapy was considerably greater in the test group.

A cross-sectional study by Bahramian, A. et al. [34] investigated the connection between recurrent aphthous stomatitis (RAS) and serum and salivary vitamin D levels. Serum vitamin D levels were significantly lower in 26 RAS patients than in 26 healthy individuals, but no statistically significant difference in the mean salivary vitamin D levels was found. Additionally, a statistically significant link between serum and salivary vitamin D levels was discovered.

### 3.5. Dental Caries

The impact of hypovitaminosis on dental caries has been the topic of three studies included in this review. One study assessed vitamin B12 hypovitaminosis, and the other two examined blood serum vitamin D levels.

Hugar, S. et al. [10] performed a cross-sectional study assessing the level of vitamin B12 and correlating it with dental caries and gingival disease. A total of 42 healthy children with consistent oral hygiene habits, no other systemic health issues, no undergoing treatment, and with similar hygienic practices overall were included. A few children followed a vegetarian diet. In total, 64% of the children showed vitamin B12 deficiency, and they had considerably more decaying, missing, and filled permanent teeth compared to the children with normal vitamin B12 levels. Additionally, children who ate a vegetarian diet had a greater rate of caries. Children who consumed a vegetarian diet exhibited a higher incidence of caries. This is due to the fact that cobalamin is predominantly present in animal-derived foods. Consequently, individuals residing in impoverished conditions or adhering to religious or personal beliefs that prohibit animal product consumption often have limited or no intake of vitamin B12.

Analysis of the relationship between blood vitamin D concentrations and dental caries was studied by Kim, I.-J. et al. [35] in a cross-sectional study that included 1688 children. The decay-missing-filled teeth index and decayed-missing-filled rate were used to subtract the incidence of caries in the permanent dentition. The group with lower levels of vitamin D had a greater incidence of caries in the permanent dentition and permanent first molar compared to the group with normal vitamin D levels. Furthermore, while the overall incidence of dental caries did not demonstrate a significant correlation with vitamin D levels, a significant association was identified, particularly regarding first molar caries, when accounting for extrinsic variables. This implies that although the direct relationship between vitamin D and caries may not be straightforward, specific tooth types, such as first molars, could exhibit heightened susceptibility to caries among individuals with lower levels of vitamin D after adjusting for external factors, including sex, household income, age, and frequency of tooth brushing.

Gyll, J. et al. [36] performed a cross-sectional study that investigated vitamin D levels and caries in children. The study recruited 8-year-old children who had participated in an intervention study on vitamin D supplementation at the age of 6. Out of 206 children from the prior study, 85 agreed to participate in an examination of their dental status. Caries and enamel defects were examined with the addition of self-reported information on dietary habits and oral behavior, and saliva was analyzed for *Streptococcus mutans* and *Streptococcus sobrinus*. Out of the remaining 85 children, 48 were caries-free, and 37 had caries. Vitamin D did not differ between children with or without caries, although the proportion with lower concentrations of vitamin D tended to be higher among children with caries. The prevalence of enamel defects on the permanent first molars and central upper and lower incisors did not differ between children with and without vitamin D insufficiency.

### 3.6. Gingival and Periodontal Disease

Hypovitaminosis as a manifestation of gingival disease has been a topic of three studies included in this review. One study examined serum vitamin C, while the other two evaluated vitamin D levels.

In a cross-sectional study, Munday, M.R. et al. [13] investigated serum vitamin C values of 20 patients diagnosed with periodontitis (9 of them stage III and 9 stage IV; 4 were smokers, 4 had diabetes, 3 had thyroid disease, 1 had Crohn’s disease, and 5 had hypertension). In total, 6 out of 20 patients had vitamin C values that were below the normal range, and 5 of those patients had stage IV periodontitis.

Proinflammatory cytokine and vitamin D levels were examined in 17 patients with rheumatoid arthritis and periodontitis (RA + P) and 18 healthy individuals before and after the initial periodontal therapy by Balci Yuce, H. et al. [27]. Blood samples, gingival crevicular fluid, and clinical periodontal measurements were taken. Vitamin D levels increased in the RA + P group compared to healthy controls, but levels dropped after periodontal therapy, suggesting that vitamin D levels may be a key predictor of periodontal bone loss.

A randomized controlled clinical trial by Woelber, J.P. et al. [26] explored the connection between an anti-inflammatory diet and gingivitis. The study involved 30 participants: 15 in the control group and 15 in the experimental group. The control group received instructions not to make any changes to their diet and oral hygiene, while the experimental group was introduced to an oral health-optimized diet. Full mouth periodontal examination was performed before and after the trial. The experimental group showed a significantly higher reduction in gingival inflammation, and bleeding on probing (BoP) dropped. There were no changes in probing pocket depth (PPD). The control group showed a significant increase in PPD and no changes in the reduction in gingival inflammation and BoP. The experimental group showed an increase in serum vitamin D.

### 3.7. Malformations

Developmental defects of enamel and vitamin deficiency were the topic of one study included in this review.

A longitudinal study by Borsting, T. et al. [31] examined the effect of maternal vitamin D and the development of enamel defects in children (mother and child paired). The maternal vitamin D exposure variables were calculated using the vitamin D measurements at the two gestational measurement points, and hypomineralized second primary molars (HSPMs) and molar incisor hypomineralization (MIH) in 176 children were examined. One-third of the participating mothers were part of the insufficiency group. A total of 39 children (22%) had HSPM, and 55 children (32%) had MIH. The average number of teeth affected was 0.45 and 0.91, respectively. There were significantly more impacted teeth in MIH patients in the low maternal vitamin D group, while HSPM patients did not differ significantly. Children with MIH who had lower levels of maternal vitamin D had more teeth affected than those with greater levels of maternal vitamin D.

### 3.8. Oral Carcinoma

One study by Nuszkiewicz, J. et al. [37] assessed vitamin D deficiency in patients with oral, lip, and pharyngeal carcinoma in situ. Patients were divided into three groups: a younger cancer group (YCG, 25 patients), an older cancer group (OCG, 50 patients), and a control group of 25 healthy individuals. The concentrations of vitamin D in the serum were considerably lower in YCG and OCG patients.

### 3.9. Perioperative Bleeding

Vitamin K as an oral anticoagulant and as an important factor in perioperative bleeding was a topic of one study included in this review. The relationship between vitamin K antagonists and the bleeding risk of invasive dental procedures was examined by Biedermann, J.S. et al. [32]. A total of 2329 procedures were performed in 1845 patients. Out of 2004 low-risk operations, 67 had clinically relevant oral bleeding within 30 days. Patients receiving antiplatelet treatment experienced oral bleeding substantially more frequently than non-users. The risk of bleeding after continuing vitamin K antagonists (VKAs) was comparable to the risk after stopping VKAs. Lower bleeding risk was linked to continued use of VKAs. In total, 294 out of 325 high-risk procedures were reported to the clinic at least 24 h in advance, and VKA therapy was stopped in 89.8% of these cases. Clinically, severe oral cavity bleeding occurred within 30 days after surgery in 6.5% of cases. Overall, compared to VKA interruption, VKA continuation was connected to a significantly increased risk of bleeding.

### 3.10. Burning Mouth Syndrome

Effects of vitamins on burning mouth syndrome were included in one study in this review. Vitamin B and zinc were used as a therapeutic regimen in a controlled clinical trial by Jankovskis, V. et al. [28]. In total, 89 patients took part in this treatment, and 20 used the capsaicin rinse. A visual analog scale (VAS) was utilized to quantify the pain or burning sensation. Stimulated and unstimulated saliva were collected during the appointments, and patients received zinc and vitamin B complex pills. Areas affected by burning sensations were the tip of the tongue (77 patients, 86.5%), sides of the tongue (56 patients, 62.9%), ventral surfaces of the tongue (30 patients, 33.7%), lips (24 patients, 26.%), and cheeks (7 patients, 7.9%). The disease lasted 6 months on average. Data showed a burning score from 2 to 4 prior to therapy and from 0 to 2 following the treatment, indicating an overall lowering of pain/burning sensation level. Unstimulated salivary flow was improved in 28 patients and stimulated in 31 patients

## 4. Discussion

This systematic review aimed to evaluate available studies regarding oral and dental expressions of hypovitaminosis and/or the therapeutic effect vitamins have in treating oral diseases.

The results of the systematic review on hypovitaminosis and its effects on oral health outcomes, when seen in the context of other data, are consistent with other studies that have demonstrated a correlation between vitamin deficiencies and a range of oral symptoms. In addition, this research highlights the importance of considering nutritional factors in oral health issues and supports the notion that specific vitamin supplementation may be beneficial in reducing certain oral health issues.

It is important to consider the limitations of the reviewed studies, such as recall bias, inadequate exposure assessment, and small sample sizes. These limitations may affect the overall reliability and generalizability of the findings. Synthesizing the results is further complicated by the variability in study designs, participant characteristics, and outcome measures. This study has certain limitations, such as the relatively small number of included studies (specifically for vitamin C, vitamin E, and vitamin K) and the utilization of cross-sectional or retrospective study designs that restrict the understanding of the long-term impacts of vitamin deficiencies on outcomes related to oral health. To advance our understanding of the role of vitamins in oral health, these limitations must be addressed through robust study designs and greater sample sizes.

Despite these challenges, this review highlights the potential role of vitamin supplementation in managing oral conditions and emphasizes the importance of addressing nutritional factors in oral health interventions.

An examination of the oral cavity aids in the early identification of nutritional deficiency and systemic illness since the oral cavity is often one of the first locations to exhibit clinical indications. The oral cavity’s anatomic structures are exposed to frequent chemical, mechanical, thermal, and viral stresses, and their rapid cell development and turnover make them vulnerable to injury when dietary deficits are present [2]. Prior to commencing the intraoral examination, it is imperative to inquire about any recent alterations in taste perception, sensations of burning, discomfort, or instances of gingival bleeding from the patient. By discerning recent changes in sensory perceptions and oral discomfort, clinicians can effectively discern between symptoms attributable to underlying pathologies and those arising from external factors, such as dietary changes, environmental exposures, or medication usage. This approach enhances diagnostic accuracy by enabling the exclusion of non-pathological contributors, thereby facilitating a more precise assessment of oral health status. Conducting an intraoral examination necessitates the identification and targeted intervention of any deficiencies present, ranging from nutritional insufficiencies to pathological conditions. Severity assessment of the deficiency aids in understanding its potential impact on oral health and overall well-being, guiding further diagnostic steps as needed, potentially involving consultation with specialists such as oral pathologists. Upon definitive diagnosis, a tailored treatment plan is devised, which may include nutritional counseling, oral hygiene instructions, medication, or specialist referral for comprehensive management. Patient education plays a pivotal role in clarifying the deficiency’s nature, implications, and recommended treatment, alongside preventative measures and post-treatment care. Follow-up appointments are scheduled to monitor treatment progress and ensure effective deficiency resolution. This systematic approach ensures thorough examination, precise diagnosis, and targeted intervention for optimal patient outcomes in oral health management [38].

This review does not cover studies on vitamin A. Infections can exacerbate vitamin A deficiency, while supplementation reduces the risk of serious illness by regulating cellular activities. Vitamin A is essential for wound healing across all stages, promoting epithelial growth, fibroblasts, granulation tissue, angiogenesis, collagen production, epithelialization, and fibroplasia [4].

There is an abundance of evidence assessing the importance of measuring blood serum vitamin B12 as well as using vitamin B12 topically or as a dietary supplement. Vitamin B12 hypovitaminosis is usually connected with pernicious and megaloblastic anemia that results in oral disease as a secondary condition. All included articles showed that the plasma vitamin B12 and folate levels are significantly lower in patients with oral efflorescence (recurrent aphthous stomatitis, atrophic glossitis, lingual linear lesions, geographic tongue, or recurrent oral ulcer). In addition, when treating patients with RAS and BMS, vitamin B12 can contribute to rapid improvement in oral signs and symptoms, such as faster healing, reduction in ulcers, disease-free periods, and reduction in pain. Most clinical guidelines recommend starting cobalamin therapy in patients with severe clinical signs of deficiency, even when the serum vitamin B12 level is within normal limits. Also, some results indicate that there is a connection between vitamin B12 deficiency in children and dental caries, which should be further investigated in a larger population. A comprehensive approach is utilized for diagnosing vitamin B deficiency, including clinical assessment, laboratory testing, and symptom consideration. When it comes to identifying vitamin B deficiencies, particularly vitamin B12 insufficiency, blood tests are crucial. Along with other markers, like hemoglobin levels and red blood cell size, these tests typically involve measuring the blood’s levels of vitamin B12 and folate. If there is a suspicion of vitamin B12 deficiency but the levels are borderline, further testing, such as identifying homocysteine or serum methylmalonic acid (MMA) levels, could potentially confirm the diagnosis. These tests assist in the distinction between folate and vitamin B12 deficiency and evaluate the beneficial effects of therapy [39]. With the exception of vegans, deficiencies in vitamin B12 are most often the result of issues with absorption and digestion rather than low consumption. Supplementation is often required to address deficiencies. A daily oral dosage of 1000 mcg has shown effectiveness equivalent to intramuscular injections once a month. Weekly intramuscular injections of 1000 mcg may be used as an initial intervention until blood levels return to normal. Oral supplements can be administered afterward for maintenance [38].

Advanced periodontal disease and elevated C-reactive protein (CRP) were both associated with low vitamin C. This discovery is likely biologically significant. Vitamin C functions as a reducing agent and displays a variety of enzymatic and non-enzymatic actions, many of which may be attributed to its ability to provide electrons. It may perform a wide range of functions. For example, it functions as a cofactor for multiple enzymes, including those that hydroxylate collagen, protect against oxidative damage to intracellular proteins and DNA, and in plasma, it promotes endothelium-dependent vasodilatation while lowering extracellular oxidants from neutrophils. Vitamin C deficiency causes scurvy, which can only be treated with vitamin C. It has been demonstrated that the plasma vitamin C levels of those with periodontitis and gingivitis are lower than those of healthy controls. Vitamin absorption is compromised in periodontitis patients [15]. A daily dose of 30–180 mg of vitamin C—or two servings of foods high in the vitamin—is recommended for maximum absorption. After this point, 50% of the intake is absorbed, with the remaining 50% being eliminated in the urine. A therapeutic dose of 1000 mg per day is administered for two weeks, after which 250 mg per week is used for maintenance [38].

Several studies agreed on the importance of serum levels of vitamin D as a predictor of periodontal bone loss and the formation of dental defects. A connection between dental caries and periodontal disease has also been made, but further examination is needed to conclude its relevance. Vitamin D serum levels were significantly lower in RAS and oral cancer patients compared to healthy individuals, which suggests that further research may be necessary to determine the impact of vitamin D on the disease. The possible correlation with RAS can be explained by the important function of vitamin D in the innate and acquired immune systems, its capacity to affect the production of proinflammatory cytokines, and the presence of dendritic cells, T- and B-lymphocytes. Also, vitamin D may induce apoptosis and inhibit invasion, cell proliferation, and tumor angiogenesis. It has anticancer action in a variety of cells [17]. Scientific organizations have developed recommendations for vitamin D supplementation and optimal serum vitamin D concentrations: target concentration of 30 ng/mL (75 nmol/L) with doses ranging from 400 to 2000 IU/day, varying by age, weight, disease status, and ethnicity [40].

Vitamin E is investigated only in terms of therapy for oral disease in various studies and reviews. The included study combined it with vitamin B; therefore, the influence of vitamin E could not be distinguished.

When vitamin K hypovitaminosis was investigated, oral bleeding was the main concern. VKA is a commonly prescribed anticoagulant and can induce vitamin K deficiency. For high-risk procedures, it is advised that VKA treatment should be interrupted and NSAIDs should be avoided.

Most forms of hypovitaminosis manifest as a nutritional deficiency in a component of the food that is consumed, and they may have favorable or unfavorable impacts on dental health [41]. Diet is crucial in preventing oral disorders such as periodontal disease, dental caries, developmental defects, and oral mucosa diseases [42]. A dental condition brought on by hypovitaminosis can develop later in various systemic disorders in addition to the dietary factor. Only strong evidence supports the impact of vitamins on dental health when both aspects are considered.

The results indicate a recurring connection between hypovitaminosis and a variety of oral symptoms, emphasizing the need for additional research to identify the underlying causative pathways. The data provided support the potential benefits of targeted vitamin supplementation in treating oral diseases despite the limitations inherent in the reviewed studies. However, robust study designs and larger sample sizes are necessary to fully understand the long-term impacts of vitamin deficiencies on oral health. Longitudinal studies could provide valuable insights into the long-term impacts of vitamin deficiencies on oral health outcomes, shedding light on causal mechanisms and potential therapeutic interventions. Exploring the effectiveness of targeted vitamin supplementation in managing oral conditions and preventing oral diseases is another avenue for future research. Additionally, investigating the interplay between nutritional factors and systemic disorders in the development of oral health issues would offer a comprehensive understanding of the complex interactions.

## 5. Conclusions

Vitamins are crucial for both the prevention and the treatment of various oral diseases. Additionally, for effective treatment and recovery, a thorough oral examination and a multidisciplinary approach are required to identify oral signs and/or symptoms and link them to diseases and vitamin-deficient conditions. Further research is needed for a better understanding of the mechanisms underlying the relationship between hypovitaminosis and oral health outcomes and to explore the effectiveness of targeted interventions in improving oral health.

## Figures and Tables

**Table 1:** PICO criteria.

Patient and population (P)	Adults
Intervention (I)	Increase in vitamin intake
Comparator or control group	Typical intake of vitamins
Outcomes (O)	Relief from hypovitaminosis symptoms

**Table 2:** PICO criteria.

Patient and population (P)	Adults
Intervention (I)	Vitamin treatment of oral diseases
Comparator or control group	Typical intake of vitamins
Outcomes (O)	Regression of oral diseases

**Table 3:** Results of risk-of-bias assessment for randomized trials, according to the current version of the Cochrane RoB 2 tool.

Study	Randomization Process	Deviations from Intended Interventions	Missing Outcome Data	Measurement of Outcome Data	Selection of the Reported Result	Overall Risk-of-Bias Judgment
Cui, J. et al. [25]	Some concerns	Some concerns	Low risk	Some concerns	Some concerns	Some concerns
Woelber, J.P. et al. [26]	Low risk	Low risk	Low risk	Low risk	Low risk	Low risk
Balci Yuce, H. et al. [27,28]	Low risk	Low risk	Low risk	Low risk	Low risk	Low risk
Jankovskis, V. et al. [29]	Low risk	Low risk	Low risk	Low risk	Some concerns	Low risk

**Table 4:** Results of risk-of-bias assessment for non-randomized trials, according to the current version of the Cochrane ROBINS-I tool.

Study	Bias due to Confounding	Bias in Selection of Participants for the Study	Bias in Classification of Interventions	Bias due to Deviations from Intended Interventions	Bias due to Missing Data	Bias in Measurement of Outcomes	Bias in Selection of the Reported Result	Overall Risk-of-Bias Judgment
Bao, Z. et al. [29]	Low risk	Low risk	Low risk	Low risk	Low risk	Low risk	Low risk	Low risk
Khayamzadeh, M. et al. [30]	Low risk	Low risk	Low risk	Low risk	Low risk	Low risk	Low risk	Low risk
Borsting, T. et al. [31]	Low risk	Low risk	Low risk	Low risk	Low risk	Low risk	Low risk	Low risk
Biedermann, J.S. et al. and Wu, Y.H. et al. [32,33]	Low risk	Low risk	Low risk	Low risk	Low risk	Low risk	Low risk	Low risk

**Table 5:** Results of risk-of-bias assessment for cross-sectional studies, according to the current version of the Newcastle–Ottawa assessment scale.

Study	Selection	Comparability	Outcome	Overall
Wu, Y.-H. et al. [33]	5	1	3	9 (High Quality)
Bahramian, A. et al. [34]	4	1	3	8 (High Quality)
Hugar, S. et al. [13]	3	1	3	7 (High Quality)
Kim, I.-J. et al. [35]	5	1	2	8 (High Quality)
Gyll, J. et al. [36]	5	1	3	9 (High Quality)
Munday, M.-R. et al. [13]	5	1	2	8 (High Quality)
Nuszkiewicz, J. et al. [37]	4	1	2	7 (High Quality)

**Table 6:** A summary of included studies.

Author	Title	Year	Type of Study	Oral Manifestation/s	Number of Participants	Age Range of Participants	Intervention/Exposure	Comparator/Control	Vitamin	Outcomes of Interest	Main Results
Wu, Y.-H. et al. [33]	Hemoglobin, iron, vitamin B12, and folic acid deficiencies and hyperhomocysteinemia in Behcet’s disease patients with atrophic glossitis	2018	Cross-sectional study	Recurrent aphthous stomatitis (RAS), atrophic glossitis (AG)	30 AG + RAS/BD patients, 33 AG־RAS/BD patients, and 126 healthy control subjects	18–82	Measurement of blood hemoglobin, iron, vitamin B12, folic acid, and homocysteine concentrations	Healthy control subjects	B9, B12	Hemoglobin, iron, vitamin B12, and folic acid deficiencies	AG + RAS/BD patients had significantly higher frequencies of hemoglobin, iron, vitamin B12, and folic acid deficiencies than healthy control subjects. They also had significantly higher frequencies of hemoglobin and vitamin B12 deficiencies than AG־RAS/BD patients. AG־RAS/BD patients had significantly higher frequencies of hemoglobin and iron deficiencies than healthy control subjects
Bao, Z. et al. [29]	Lingual Linear Lesions: A Clinical Sign Strongly Suggestive of Severe Vitamin B12 Deficiency	2020	Retrospective study	Lingual linear lesion (LLL)	57 patients presenting with lingual linear lesions (LLLs)	18–80	Measurement of serum levels of vitamin B12, folate, ferritin, hemoglobin, and zinc	/	B12	Serum levels of vitamin B12, folate, ferritin, hemoglobin, and zinc	98.25% of patients had severe serum vitamin B12 deficiency. LLLs were associated with significantly lower mean levels of vitamin B12 and significantly higher mean MCV, rapid improvement after replacement therapy
Khayamzadeh, M. et al. [30]	Determining salivary and serum levels of iron, zinc and vitamin B12 in patients with geographic tongue	2019	Case–control study	Geographic tongue	20 patients with geographic tongue, 20 healthy individuals	19–49	Evaluation of iron, zinc, and vitamin B12 levels in blood and saliva	Healthy subjects without geographic tongue	B12	Salivary and serum levels of iron, zinc, and vitamin B12	Patients with geographic tongue had lower salivary zinc levels compared to the control group. No significant differences were found in serum levels of iron, zinc, and vitamin B12
Cui, J. et al. [25]	Clinical Evaluation and Therapeutic Effects of Combination Treatment withMecobalamin + Vitamin E in Recurrent Oral Ulcer	2022	Randomized controlled trial	Recurrent oral ulcer	58 patients with aphthous oral ulcers (29 in the experimental group, 29 in the control group)	45–50	Mecobalamin tablets + vitamin E	Fe complex enzyme gargle	B12	Pain level (VAS score), ulcer status and number, proinflammatory cytokine levels, quality of life (SF-36), mean intermission time, mean ulcer healing time, and total effectiveness rate	Longer total intermission time, shorter mean ulcer healing time, fewer total ulcers, lower pain scores in test group, improved QoL in test group, and lower proinflammatory cytokine levels in test group
Hugar, S. et al. [10]	Assessment of Vitamin B12 and Its Correlation with Dental Caries and Gingival Diseases in 10- to 14-year-old Children: A Cross-sectional Study	2017	Cross-sectional study	Dental caries and gingival disease	42 healthy children (21 boys, 21 girls)	10–14	Serum vitamin B12 levels	/	B12, E	Dental caries (DMFT score) and gingival diseases (PI, GI scores)	64% of children had vitamin B12 deficiency, higher DMFT scores in deficient children, negative correlation between vitamin B12 levels and dental caries, and gingival indices
Jankovskis, V. et al. [28]	Vitamin B and Zinc Supplements and Capsaicin Oral Rinse Treatment Options for Burning Mouth Syndrome	2021	Randomized controlled study	Burning mouth syndrome (BMS)	89 patients (BMS), 20 patients (capsaicin rinse)	Not provided Mean age: 59 ± 19 years	Vitamin B complex and zinc supplements; 0.02% topical capsaicin rinse	/	B12	Pain/burning levels (assessed via visual analog scale) and salivary flow	Both treatment methods showed statistically significant reductions in pain/burning levels and no significant changes in salivary flow
Munday, M.-R. et al. [13]	A Pilot Study Examining Vitamin C Levels in Periodontal Patients	2020	Cross-sectional study	Periodontal disease	20 patients with periodontitis	Not provided Mean age: 65 ± 9	Serum vitamin C and C-reactive protein (CRP)	/	C	Serum vitamin C levels, C-reactive protein (CRP) levels, periodontal disease stage, and dietary intake	6 out of 20 patients had vitamin C levels below the institutional normal range, low vitamin C was associated with higher periodontal disease stage, elevated CRP was found in 2/3 of individuals with low vitamin C, with a significant negative correlation between vitamin C and CRP, and vitamin C levels did not correlate with patient-reported fruit or vegetable consumption but were associated with high processed meat intake
Woelber, J. P. et al. [26]	The influence of an anti-inflammatory diet on gingivitis. A randomized controlled trial	2019	Randomized controlled trial	Gingivitis	30 participants (15 in the experimental group, 15 in the control group)	Not provided Mean age of experimental group: 27,3 ± 4,7 years Mean age of control group: 33,7 ± 1	Diet low in processed carbohydrates and animal proteins, rich in omega-3 fatty acids, vitamin C, vitamin D, antioxidants, plant nitrates, and fibers for 4 weeks. Both groups suspended inter-dental cleaning	No change in diet. Both groups suspended inter-dental cleaning	C, D	Gingival bleeding (GI), plaque index (PI), probing pocket depths, bleeding on pocket probing, periodontal inflamed surface areas (PISA), body mass index, weight, and subgingival microbiome composition	Experimental group showed significant reduction in gingival bleeding (GI), significant increase in vitamin D values, and significant weight loss. No significant differences in plaque values between groups. No significant differences in inflammatory serological parameters or subgingival microbiome composition between groups
Bahramian, A. et al. [34]	Comparing Serum and Salivary Levels of Vitamin D in Patients with Recurrent Aphthous Stomatitis and Healthy Individuals	2018	Cross-sectional study	Recurrent aphthous stomatitis (RAS)	26 RAS patients, 26 healthy individuals	18–60	Evaluation of serum and salivary vitamin D levels	Healthy Individuals	D	Serum and salivary levels of vitamin D	Serum vitamin D levels were significantly lower in patients with RAS compared to healthy individuals. Salivary vitamin D levels did not show a significant difference between patients with RAS and healthy individuals. A significant positive correlation was observed between serum and salivary levels of vitamin D in all participants
Kim, I.-J. et al. [35]	A cross-sectional study on the association between vitamin D levels and caries in the permanent dentition of Korean children	2018	Cross-sectional study	Dental caries	1688 children	10–12	Blood vitamin D [25(OH)D] concentrations	/	D	Association between 25(OH)D levels and dental caries	The group with 25(OH)D levels < 50 nmol/L had higher proportion of caries in permanent dentition and first molar, no significant correlation between 25(OH)D levels and caries when controlling for external factors, but significant correlation with first molar caries. Children with 25(OH)D levels < 50 nmol/L 1.295 times more likely to have first molar caries and negative correlation between 25(OH)D levels and DMFT
Gyll, J. et al. [36]	Vitamin D status and dental caries in healthy Swedish children	2018	Cross-sectional study	Dental caries	85 children	8	Vitamin D supplementation	/	D	Dental caries, enamel defects, saliva LL37 levels	Weak inverse association between vitamin D status at 6 years and caries 2 years later. Higher vitamin D levels correlated with being caries-free. Vitamin D status unrelated to enamel defects but positively associated with saliva LL37 levels
Balci Yuce, H. et al. [27]	Assessment of local and systemic 25-hydroxy-vitamin D, RANKL, OPG, and TNF levels in patients with rheumatoid arthritis and periodontitis	2017	Randomized controlled trial	Periodontal disease	17 RA + CP patients, 18 CP patients, 18 healthy controls	RA + CP patient: 37–61 CP patients: 36–65. Control group: 36–65	Nonsurgical periodontal treatment	Healthy individuals	D	Clinical periodontal parameters, GCF, and serum levels of vitamin D	GCF vitamin D levels were higher in RA +CP and CP groups than in healthy controls but decreased in the RA + CP group after periodontal treatment. Local vitamin D levels might be an important indicator of periodontal bone loss
Borsting, T. et al. [31]	Maternal vitamin D status in pregnancy and molar incisor hypomineralisation and hypomineralised second primary molars in the offspring at 7–9 years of age: a longitudinal study	2022	Longitudinal study	Molar incisor hypomineralization (MIH) and hypomineralized second primary molars (HSPM)	176 mother and child pairs	7–9	Maternal serum 25-hydroxyvitamin D levels	/	D	Molar incisor hypomineralization (MIH) and hypomineralized second primary molars (HSPM)	Insufficient maternal serum vitamin D at mid-pregnancy was associated with a higher number of affected teeth among the offspring with MIH at 7–9 years of age. Further prospective studies are needed to investigate whether this finding is replicable and to clarify the role of maternal vitamin D status during pregnancy and MIH, as well as HSPM, in children
Nuszkiewicz, J. et al. [37]	Parameters of Oxidative Stress, Vitamin D, Osteopontin, and Melatonin in Patients with Lip, Oral Cavity, and Pharyngeal Cancer	2021	Cross-sectional study	Lip, oral cavity, or pharyngeal cancer in situ	25 LOCP patients (YCG), 20 LOCP elderly patients (OCG), 25 healthy volunteers	Not provided Mean age of YCG: 58:24 ± 1:29. Mean age of OCG: 69:7 ± 1:49. Mean age of control group: 55:36 ± 1:17	Concentrations of vitamin D, osteopontin, melatonin, and malondialdehyde; activities of antioxidant enzymes	Healthy volunteers	D	Vitamin D deficiency and disturbed oxidant–antioxidant homeostasis observed in LOCP patients; association of osteopontin with LOCP carcinogenesis	Disruption of oxidant–antioxidant homeostasis in the lip, oral cavity, and pharyngeal cancer patients impaired antioxidant enzymatic defense, and increased lipid peroxidation, correlated with high levels of osteopontin, was determined in this type of cancer. However, vitamin D deficiency in the LOCP patients was found
Biedermann, J.S. et al. [32]	Predictors of oral cavity bleeding and clinical outcome after dental procedures in patients on vitamin K antagonists	2017	Cohort study	Periprocedural bleeding	1845 patients	64–81	VKA management strategies (continuation with tranexamic acid mouthwash, interruption with or without bridging)	/	K	Clinically relevant oral cavity bleeding, non-oral cavity bleeding, thromboembolic events, hospitalization, all-cause mortality within 30 days	Bridging therapy, antiplatelet therapy, and a supratherapeutic or unobjectified INR before the procedure were identified as strongest predictors of oral cavity bleeding. Different management strategies showed varying risks of oral cavity bleeding

## Data Availability

The data presented in this article are available on request from the corresponding authors.
